# A Bazilevic-type function associated with the class of univalent functions with study Coefficient Bounds and Fekete-Szego inequalities

**DOI:** 10.12688/f1000research.172490.2

**Published:** 2026-09-15

**Authors:** Mays S.Abdul Ameer, Abdul Rahman S.Juma, Hassan Hussien Ebrahim

**Affiliations:** 1 Mathematics, Tikrit University, Tikrit, Saladin Governorate, Iraq; 2 Mathematics, University of Anbar, Ramadi, Al Anbar Governorate, Iraq; 3 Mathematics, Tikrit University, Tikrit, Saladin Governorate, Iraq

**Keywords:** Analytic function, Univalent functions, Diﬀerential operator, Subordination, Bazilevic type, Fekete-Szego inequalities.

## Abstract

**Background:**

The Fekete–Szegö inequality and coefficient bounds play a fundamental role in the study of Bazilevič-type functions, a subclass of univalent functions with significant applications in complex analysis and related mathematical fields. These functions have been extensively studied for their geometric properties and coefficient behavior, yet there remains a need to explore subclasses defined via differential operators and subordination techniques to obtain sharper and more general bounds. Understanding the coefficient structure of these functions provides insight into their analytic behavior and extends classical results in the theory of univalent functions.

**Methods:**

This study focuses on a specific subclass of Bazilevič-type functions and investigates its properties using differential subordination and differential operators. These mathematical tools are employed to derive explicit coefficient estimates and establish the Fekete–Szegö inequalities. The analysis involves careful application of operator techniques to obtain bounds that reflect the influence of the subclass parameters on the analytic functions under consideration.

**Results:**

The derived inequalities demonstrate the effectiveness of differential operators in obtaining precise coefficient constraints. The results highlight how variations in the defining parameters of the subclass influence the function behavior, providing clear and explicit bounds for the coefficients. These findings extend existing results in the literature and offer new insights into the structure of Bazilevič-type functions.

**Conclusions:**

Overall, this study provides a systematic approach to understanding the coefficient structure of Bazilevič-type functions. The findings establish a foundation for further theoretical research on univalent functions and offer tools that can be applied in both theoretical investigations and practical problems in complex analysis. The results contribute to the broader understanding of analytic functions and their applications in mathematical modeling.

## Introduction

Geometric function theory is a subfield of complex analysis that studies the geometric characteristics of analytic functions. The foundation of complex analysis is academic research on univalent and multivalent function theory. find it fascinating because of its complex geometry and variety of research options. Understanding univalent functions is crucial for the complicated analysis of single and multiple variables. Univalent functions, which are analytic and one-to-one in the unit disk, form a fundamental class in complex analysis and geometric function theory, with numerous applications in mathematical modeling and theoretical investigations. ^1^^–^^3^ Among the various subclasses, Bazilevič-type functions, introduced by Bazilevich, ^4^^–^^7^ have drawn significant attention due to their rich geometric properties and connections with differential subordination. ^8^ These functions have been widely studied for their coefficient bounds and associated inequalities, including the classical Fekete–Szegö problem, which provides important constraints on the coefficients of analytic and multivalent functions. ^9^^–^^12^ Recent developments have focused on extending these studies to subclasses defined via differential operators and linear transformations, allowing for the derivation of sharper coefficient estimates and Fekete–Szegö inequalities. ^13^^,^^14^ Investigations on bi-Bazilevič functions, Faber polynomial coefficients, and operator-based approaches have further highlighted the effectiveness of these methods in obtaining explicit bounds and revealing structural properties of analytic functions. ^15^ Despite these advances, there remains a need to explore new subclasses of Bazilevič-type functions associated with specific differential or linear operators, particularly in relation to their coefficient estimates and Fekete–Szegö inequalities. ^16^^–^^18^ This study addresses this gap by examining the subclass, establishing explicit coefficient bounds, and formulating relevant Fekete–Szegö inequalities. The findings provide a systematic framework for understanding the analytic and geometric properties of Bazilevič-type functions and offer potential directions for future research in the theory of univalent functions.

## Methods

Consider the open unit disc to be Ω={z:|z|<1} and let *D* stand for the class of analytic functions of the type

$$f\left(z\right)=z+\sum_{n=2}^{\infty }a_{n}z^{n}.$$ (1)

The functions are normalized with $f\left(0\right)=0\;\text{and}\;f^{\prime }\left(0\right)=1,$ and they are analytic in the open unit disk.

In the open unit disk Ω , let E represent the subclass of all univalent functions.

A refers to the class of functions h(z) with a positive real fraction in Ω . As follows.

$$h\left(z\right)=1+\sum_{n=1}^{\infty }c_{n}z^{n}.$$

Since $w\left(z\right)=\bar{z\;}$ is an analytic function on ∂D, we define w(z) as the Schwarz function defined on the domain's border ∂D in ℂ . ^1^^,^^2^

The class A has the following relationship with the class of Schwarz functions w see Ref. 3.

$$h\in A↔h=\frac{1+w}{1-w}$$ (2)

Consider two analytic functions in Ω , *f* and *h*, then we say f(z) is subordinate to g(z) . Write as follows.

f≺gorf(z)≺g(z),(z∈Ω), (3)

If there is an analytic Schwarz function in Ω with w(0)=0 and |w(z)|<1, then

f(z)=g(w(z)). If a function g(z) be univalent in Ω , then f(z)≺g(z)i s equivalent to f(0)=g(0)andf(Ω)⊂g(Ω) (see Ref. 4).

If a function f(z) meets the following criteria, it is referred to as univalent starlike in Ω :

$$\text{Re}\left\{\frac{z\overset{´}{f\left(z\right)}}{f\left(z\right)}\right\}>0,\left(z\in \Omega \right).$$

$S^{*}$ is the name given to the old class of functions (see Ref. 3). We can write from Equation (1)

$${\left(f\left(z\right)\right)}^{\beta }={\left(z+\sum_{n=2}^{\infty }a_{n}z^{n}\right)}^{\beta },$$ (4)

If β is a real number greater than zero, we can obtain

$$\begin{array}{c} {\left(f\left(z\right)\right)}^{\beta }={\left(z+a_{2}\;z^{2}+a_{3}\;z^{3}+a_{4}\;z^{4}+\ldots \right)}^{\beta }.\\ {\left(f\left(z\right)\right)}^{\beta }=z^{\beta }{\left(1+a_{2}\;z+a_{3}\;z^{2}+a_{4}\;z^{3}+\cdots \right)}^{\beta }. \end{array}$$ (5)

By applying Equation (5) binomial expansion, we get

$${\left(f\left(z\right)\right)}^{\beta }=z^{\beta }\left[1+\beta \left(a_{2}\;z+a_{3}\;z^{2}+a_{4}\;z^{3}+\cdots \right)+\frac{\beta \left(\beta -1\right)}{2!}{\left(a_{2}\;z+a_{3}\;z^{2}+a_{4}\;z^{3}+\cdots \right)}^{2}+\ldots \right]$$

$${\left(f\left(z\right)\right)}^{\beta }=z^{\beta }(1+\beta \left(a_{2}\;z+a_{3}\;z^{2}+a_{4}\;z^{3}+\cdots \right).$$

Therefore

$${\left(f\left(z\right)\right)}^{\beta }=z^{\beta }+\beta a_{2}z^{\left(\beta +1\right)}+\beta a_{3}\;z^{\left(\beta +2\right)}+\beta a_{4}\;z^{\left(\beta +3\right)}+\cdots .$$

The following represents the class of analytic functions $D_{\beta }:$

$${\left(f\left(z\right)\right)}^{\beta }=z^{\beta }+\sum_{n=2}^{\infty }a_{n}\left(\beta \right)z^{\beta +n-1}.$$ (6)

$G\left(D_{\beta }\right)$ is the differential operator determined for a function ${\left(f\left(z\right)\right)}^{\beta }$ given in Equation (6) on the space of analytic functions.

Let $f\;\in \;G\left(D_{\beta }\right),$ where ${\left(f\left(z\right)\right)}^{\beta }=z^{\beta }+\sum_{n=2}^{\infty }a_{n}\left(\beta \right)z^{\beta +n-1}.$

Define the linear operator

$$H_{\xi ,\mu }^{m,\lambda }:G\left(D_{\beta }\right)⟶G\left(D_{\beta }\right)$$

recursively by

$$H_{\xi ,\mu }^{0,\lambda }\left({\left(f\left(z\right)\right)}^{\beta }\right)={\left(f\left(z\right)\right)}^{\beta },$$

and

$$H_{\xi ,\mu }^{m,\lambda }\;({\left(f\left(z\right)\right)}^{\beta }=H_{1}\;((H_{\xi ,\mu )}^{\left(m-1,\lambda \right)}\left({\left(f\left(z\right)\right)}^{\beta }\right),m\;\in \;\mathbb{N},$$

where the auxiliary operator $H_{1}$ is defined by

$$H_{1}\left({\left(f\left(z\right)\right)}^{\beta }\right)=\frac{1+\left(1-\beta \right)\left(\lambda -\xi \right)}{\mu +\lambda }z^{\beta }+\sum_{n=2}^{\infty }{\frac{1+\left(\lambda -\xi \right)\left(\beta +n-2\right)}{\mu +\lambda }}^{2}a_{n}\left(\beta \right)z^{\beta +n-1}.$$

Hence,

$$H_{\xi ,\mu }^{1,\lambda }\left({\left(f\left(z\right)\right)}^{\beta }\right)=H_{1}\left(H_{\xi ,\mu }^{0,\lambda }\left({\left(f\left(z\right)\right)}^{\beta }\right)\right)=H_{1}\left[z^{\beta }+\sum_{n=2}^{\infty }a_{n}\left(\beta \right)z^{\beta +n-1}\right]$$

$$H_{\xi ,\mu }^{1,\lambda }\left({\left(f\left(z\right)\right)}^{\beta }\right)={\left(\frac{1+\left(1-\beta \right)\left(\lambda -\xi \right)}{\mu +\lambda }\right)}^{1}z^{\beta }+\sum_{n=2}^{\infty }{\left(\frac{1+\left(n-\beta \right)\left(\lambda -\xi \right)}{\mu +\lambda }\right)}^{1}a_{n}\left(\beta \right)z^{\beta +n-1}$$ (7)

Similarly,

$$H_{\xi ,\mu }^{2,\lambda }\left({\left(f\left(z\right)\right)}^{\beta }\right)=H_{1}\left(H_{\xi ,\mu }^{1,\lambda }\left({\left(f\left(z\right)\right)}^{\beta }\right)\right)={\left(\frac{1+\left(1-\beta \right)\left(\lambda -\xi \right)}{\mu +\lambda }\right)}^{2}z^{\beta }+\sum_{n=2}^{\infty }{\left(\frac{1+\left(\lambda -\xi \right)\left(\beta +n-2\right)}{\mu +\lambda }\right)}^{2}a_{n}\left(\beta \right)z^{\beta +n-1}.$$

In general, we have

$$H_{\xi ,\mu }^{m,\lambda }\left({\left(f\left(z\right)\right)}^{\beta }\right)={\left(\frac{1+\left(1-\beta \right)\left(\lambda -\xi \right)}{\mu +\lambda }\right)}^{m}z^{\beta }+\sum_{n=2}^{\infty }{\left(\frac{1+\left(\lambda -\xi \right)\left(\beta +n-2\right)}{\mu +\lambda }\right)}^{m}a_{n}\left(\beta \right)z^{\beta +n-1}.$$ (8)

where $\left(\mu >0,\xi ,\lambda \;\geq \;0,m\;\in \;N_{0}=N\cup \left\{0\right\};\beta >0,z\;\in \;\Omega \right).$

The goal of this study is to find “coefficient bounds and Fekete-Szego inequalities for the subclass $L_{\xi ,\mu }^{m,\lambda }\left(\delta ,\alpha ,\gamma \right)$ of Bazilevic type functions”.

Now, using the differential operator, define a class of Bazilevic functions (see Ref. 5) as follows:

Definition 1. The function $f{\left(z\right)}^{\beta }$ which has the form Equation (5) belongs to the class $L_{\xi ,\mu }^{m,\lambda }\left(\delta ,\alpha ,\gamma \right)$ satisfies the following conditions

$$\text{Re}\left\{\frac{H_{\xi ,\mu }^{m,\lambda }f{\left(z\right)}^{\beta }}{{\left(\frac{1+\left(1-\beta \right)\left(\lambda -\xi \right)}{\mu +\lambda }\right)}^{m}z^{\beta }}\right\}>\gamma \left|\frac{H_{\xi ,\mu }^{m,\lambda }f{\left(z\right)}^{\beta }}{{\left(\frac{1+\left(1-\beta \right)\left(\lambda -\xi \right)}{\mu +\lambda }\right)}^{m}z^{\beta }}-1\right|+\alpha .$$ (9)

Where $\left(\mu >0,\xi ,\lambda ,\gamma \;\geq \;0,m\;\in \;N_{0}=N\cup \left\{0\right\};\beta >0,z\;\in \;\Omega \right)$ (see Ref. 6).

The above condition is equivalent to

$$\frac{H_{\xi ,\mu }^{m,\lambda }f{\left(z\right)}^{\beta }}{{\left(\frac{1+\left(1-\beta \right)\left(\lambda -\xi \right)}{\mu +\lambda }\right)}^{m}z^{\beta }}\;≺\;\gamma \left(z\right),$$ (10)

where $\gamma \left(z\right)=\frac{1+\mathrm{\alpha z}}{1-\mathrm{\delta \alpha z}}$ be univalent function with γ(0)=1 and $\overset{´}{\;\gamma \;}\left(z\right)>0$ .

If ξ=δ=0 and m=λ=μ=α=1, we derive the following definition of the subclass of Bazilevic univalent functions. ^4^

$$|f\;´\left(z\right){\left(\frac{f\left(z\right)}{z}\right)}^{\beta -1}|<1.$$ (11)

If ξ=0 and $m=\lambda =\mu =1,L_{0,1}^{1,1}\left(\delta ,\alpha ,\gamma \right)\left(f{\left(z\right)}^{\beta }\right)$

$$\overset{´}{f}\left(z\right){\left(\frac{f\left(z\right)}{z}\right)}^{\beta -1}\;≺\;\gamma \left(z\right).$$ (12)

If ξ=0,m=λ=β=1 and placing

$$\begin{array}{c} \gamma \left(z\right)=\frac{1+z}{1-z},\\ \overset{´}{f}\left(z\right)≺\frac{1+z}{1-z}. \end{array}$$

Bazilevic was generalized to provide the class described in (9) (see Ref. 7). Where he presented this job and studied it as follows:

$$f\left(z\right)={\left\{\frac{\xi \;}{1+\varepsilon^{2}\;}\int_{0}^{z}\left(h\left(v\right)-\mathrm{i\varepsilon}\right)v^{-\left(1+\frac{\;\mathrm{i\xi \varepsilon}}{1+\varepsilon^{2}\;}\right)}g{\left(v\right)}^{\frac{\xi \;}{1+\varepsilon^{2}}}\;\mathrm{dv}\right\}}^{\frac{1+\mathrm{i\varepsilon}\;}{\xi }},$$ (13)

where the function h(v) belongs to A and $g\left(v\right)\in S^{*}$ with β>0.

The following lemmas are necessary for you to discuss the main results.

Lemma 2. Let $h\left(z\right)=1+c_{1}z+c_{2}z^{2}+c_{3}z^{3}+\ldots ,$ z∈Ω belongs to the class A and μ∈C , then

$\left|c_{2}-\mu {c_{1}}^{2}\right|\leq 2\max\left\{1,|2\mu -1|\right\}.$ The following functions can produce crisp results. ^8^

$$h\left(z\right)=\frac{1+z^{2}}{1-z^{2}}\;\text{and}\;h\left(z\right)=\frac{1+z}{1-z},z\;\in \;\Omega .$$

Lemma 3. Let the function h(z) is analytic in *Ω*, with |h(z)|<1 and let

$$h\left(z\right)=c_{0}+c_{1}z+c_{2}z^{2}+c_{3}z^{3}+\cdots .$$

Then

$$\left|c_{0}\leq 1\right|\;\text{and}\;\left|c_{k}\right|\leq 1-{\left|c_{0}\right|}^{2}\;\text{for}\;k>0.$$

Lemma 4. If $h\left(z\right)=1+c_{1}z+c_{2}z^{2}+c_{3}z^{3}+\cdots ,z\in \Omega \;$ belongs to the class A then

$$\left|c_{2}-\gamma {c_{1}}^{2}\right|\leq \left\{ \begin{array}{ll} -4\gamma +2 & \text{if}\;\gamma \;\leq \;0\\ \;2 & \text{if}\;0\;\leq \;\gamma \;\leq \;1\\ \;4\gamma -2 & \text{if}\;\gamma \;\geq \;0. \end{array}\right.$$

For γ<0 or γ>1, equality holds if and only if h(z) equals $\frac{1+z}{1-z}$ or one of them is a rotation. If 0<γ<1, then equality holds if and only if h(z) is equal to $\frac{1+z^{2}}{1-z^{2}}$ or one of them is rotations. Inequality becomes equality when γ=0 if and only if $h\left(z\right)=\left(\frac{1+\lambda }{2}\right)$ $\frac{1+z}{1-z}+\left(\frac{1-\lambda }{2}\right)$ $\frac{1+z}{1-z}$ ,

One of its rotations, or 0≤γ≤1 . If and only if the function h is the reciprocal of one of the functions such that equality holds in the case of γ=0, equality holds for γ=1.

If the highest limit stated above is sharp, it can be made better when 0<γ<1 :

$$\left|c_{2}-\gamma {c_{1}}^{2}\right|+\gamma {\left|c_{1}\right|}^{2}\leq 2\;0<\gamma \leq \frac{1}{2},$$

and

$$\left|c_{2}-\gamma {c_{1}}^{2}\right|+\left(1-\gamma \right){\left|c_{1}\right|}^{2}\leq 2\;\frac{1}{2}\leq \gamma <1.$$

Many writers have examined Bazilevic functions, including Refs. 5, 10– 13 and 14. Studied Singh in 1973, “two subclasses of the class B-the class of Bazilevic functions”. ^15^ In 2020, Particle Umar et al. investigated a subclass related to Bazilevic functions. ^16^ Juma and AL-khafaj 2019 The characteristics and properties of a family of Bazilevic (type) functions that are provided by particular linear operators. ^17^ Faber polynomial coefficient estimates for a subclass of analytic Bi-Bazilevic functions defined by a differential operator were introduced in Refs. 6, 18.

## Results and discussion

Theorem 1. Let ${\left(f\left(z\right)\;\right)}^{\beta }\in D_{\beta }$ which is given in Equation (6). If ${\left(f\left(z\right)\;\right)}^{\beta }$ belongs to the class $L_{\xi ,\mu }^{m,\lambda }\left(\delta ,\alpha ,\gamma \right).$ Then

$$\begin{array}{c} \left|a_{2}\left(\beta \right)\right|\leq \frac{\alpha \left(1+\delta \right)}{2\alpha {\left(\frac{1+\left(\lambda -\xi \right)\left(-1-\beta \right)}{1+\left(\lambda -\xi \right)\left(1-\beta \right)}\right)}^{m}},\\ \left|a_{3}\left(\beta \right)\right|\leq \frac{\alpha \left(1+\delta \right)}{2\alpha {\left(\frac{1+\left(\lambda -\xi \right)\left(-2-\beta \right)}{1+\left(\lambda -\xi \right)\left(1-\beta \right)}\right)}^{m}\left[1-\frac{\left(1-\delta \alpha \right)A_{1}+\alpha \left(1+\delta \right)A_{3}}{2A_{1}}\right]} \end{array}$$

and

$$\begin{array}{c} \left|a_{4}\left(\beta \right)\right|\leq \frac{\alpha \left(1+\delta \right)}{2\beta {\left(\frac{1+\left(\lambda -\xi \right)\left(-3-\beta \right)}{1+\left(\lambda -\xi \right)\left(1-\beta \right)}\right)}^{m}}\\ \left[1-\frac{\left(\beta -1\right)A_{3}+2\left(1-\delta \alpha \right)A_{2}}{2A_{2}}+\frac{3\left(\beta -1\right)\left(1-\delta \alpha \right)A_{1}A_{4}+3\alpha \left(\beta -1\right)\left(1+\delta \right)A_{3}A_{4}}{12A_{1}A_{2}}-\frac{\alpha \left(\beta -2\right)\left(1+\delta \right)A_{3}A_{4}}{12A_{1}A_{2}}-\frac{3}{12}\left(1-2\delta \alpha \right)\right] \end{array}$$

where

$$\begin{array}{l} A_{1}=2\beta {\left(\frac{1+\left(\lambda -\xi \right)\left(-1-\beta \right)}{1+\left(\lambda -\xi \right)\left(1-\beta \right)}\right)}^{2m},\\ A_{2}=\beta {\left(\frac{1+\left(\lambda -\xi \right)\left(-1-\beta \right)}{1+\left(\lambda -\xi \right)\left(1-\beta \right)}\right)}^{m}{\left(\frac{1+\left(\lambda -\xi \right)\left(-2-\beta \right)}{1+\left(\lambda -\xi \right)\left(1-\beta \right)}\right)}^{m},\\ \begin{array}{c} A_{3}=\left(\beta -1\right){\left(\frac{1+\left(\lambda -\xi \right)\left(-2-\beta \right)}{1+\left(\lambda -\xi \right)\left(1-\beta \right)}\right)}^{m},\\ A_{4}=\alpha \left(1+\delta \right){\left(\frac{1+\left(\lambda -\xi \right)\left(-3-\beta \right)}{1+\left(\lambda -\xi \right)\left(1-\beta \right)}\right)}^{m}. \end{array} \end{array}$$

Proof: If ${\left(f\left(z\right)\;\right)}^{\beta }\;\in \;L_{\xi ,\mu }^{m,\lambda }\left(\delta ,\alpha ,\gamma \right),$ then from Equation (10) there is the Schwarz function w(z) . This is analytic in Ω, with w(0)=0 and |w(z)|<1 such that

$$\frac{H_{\xi ,\mu }^{m,\lambda }f{\left(z\right)}^{\beta }}{{\left(\frac{1+\left(1-\beta \right)\left(\lambda -\xi \right)}{\mu +\lambda }\right)}^{m}z^{\beta }}≺\gamma \left(w\left(z\right)\right),$$ (14)

where

$$\gamma \left(z\right)=\frac{1+\mathrm{\alpha z}}{1-\mathrm{\delta \alpha z}}=1+\alpha \left(1+\delta \right)z+\delta \alpha^{2}\left(1+\delta \right)z^{2}+\delta^{2}\alpha^{3}\left(1+\delta \right)z^{3}+\cdots .$$ (15)

The definition of the function h(z) is:

$$h\left(z\;\right)=\frac{1+w\left(z\right)}{1-w\left(z\right)}=1+c_{1}h+c_{2}h^{2}+c_{3}h^{3}+\cdots .$$

As may be seen from Equation (14), h∈A and

$$w\left(z\;\right)=\frac{1-w\left(z\right)}{1+w\left(z\right)}=\frac{1}{2}c_{1}z+\frac{1}{2}\left(c_{2}-\frac{1}{2}{c_{1}}^{2}\right)z^{2}+\frac{1}{2}\left(c_{3}-c_{1}c_{2}+\frac{1}{4}{c_{1}}^{3}\right)z^{3}+\cdots .$$ (16)

Considering Equations (14), (15), and (16), we get at

$$\begin{array}{l} \frac{H_{\xi ,\mu }^{m,\lambda }f{\left(z\right)}^{\beta }}{{\left(\frac{1+\left(1-\beta \right)\left(\lambda -\xi \right)}{\mu +\lambda }\right)}^{m}z^{\beta }}≺\gamma \left(w\left(z\right)\right)\\ =\gamma \left(z\right)\left(\frac{h\left(z\right)-1}{h\left(z\right)+1\;}\right).\\ =\gamma \left(\frac{1}{2}c_{1}z+\frac{1}{2}\left(c_{2}-\frac{1}{2}{c_{1}}^{2}\right)z^{2}+\frac{1}{2}\left(c_{3}-c_{1}c_{2}+\frac{1}{4}{c_{1}}^{3}\right)z^{3}+\cdots \right),\\ =1+\frac{1}{2}\alpha \left(1+\delta \right)c_{1}z+\left[\frac{1}{2}\alpha \left(1+\delta \right)\left(c_{2}-\frac{1}{2}{c_{1}}^{2}\right)+\frac{1}{4}\delta \alpha^{2}\left(1+\delta \right){c_{1}}^{2}\right]z^{2}+\\ +\left[\frac{1}{2}\alpha \left(1+\delta \right)(\left(c_{3}-c_{1}c_{2}+\frac{1}{4}{c_{1}}^{3}\right)+\frac{1}{2}\delta \alpha^{2}\left(1+\delta \right)\left(c_{2}-\frac{1}{2}{c_{1}}^{2}\right)c_{1}+\frac{1}{8}\delta^{2}\alpha^{2}\left(1+\delta \right){c_{1}}^{2}\right]\ldots \end{array}$$ (17)

By use of the series expansion of

$$1+\sum_{n=2}^{\infty }{\left(\frac{1+\left(\lambda -\xi \right)\left(\beta +n-1\right)}{1+(\left(\lambda -\xi \right)\left(1-\beta \right)}\right)}^{m}a_{n}\left(\beta \right)z^{n-1},$$

we have

$$\begin{array}{c} \frac{H_{\xi ,\mu }^{m,\lambda }f{\left(z\right)}^{\beta }}{{\left(\frac{1+\left(1-\beta \right)\left(\lambda -\xi \right)}{\mu +\lambda }\right)}^{m}z^{\beta }}=1+\beta {\left(\frac{1+\left(\lambda -\xi \right)\left(-1-\beta \right)}{1+\left(\lambda -\xi \right)\left(1-\beta \right)}\right)}^{m}a_{2}\left(\beta \right)\;z+\left[\beta a_{3}\;\left(\beta \right)\frac{\beta \left(\beta -1\right)}{2!}{a_{2}}^{2}\left(\beta \right)\right]{\left(\frac{1+\left(\lambda -\xi \right)\left(-2-\beta \right)}{1+\left(\lambda -\xi \right)\left(1-\beta \right)}\right)}^{m}z^{2}\\ +\left[\beta a_{4}\;\left(\beta \right)+\beta \left(\beta -1\right)a_{2}\left(\beta \right)a_{3}\left(\beta \right)+\frac{\beta \left(\beta -1\right)\left(\beta -2\right)}{3!}{a_{2}}^{3}\left(\beta \right)\right]{\left(\frac{1+\left(\lambda -\xi \right)\left(-3-\beta \right)}{1+\left(\lambda -\xi \right)\left(1-\beta \right)}\right)}^{m}z^{3}+\ldots . \end{array}$$ (18)

Based on Equations (17) and (18), we compare the coefficients of $z,z^{2}$ , and $z^{3}\;$ in Equation (14) to obtain

$$a_{2}\left(\beta \right)=\frac{\alpha \left(1+\delta \right)c_{1}}{2\alpha {\left(\frac{1+\left(\lambda -\xi \right)\left(-1-\beta \right)}{1+\left(\lambda -\xi \right)\left(1-\beta \right)}\right)}^{m}},$$ (19)

$$a_{3}\left(\beta \right)\leq \frac{\alpha \left(1+\delta \right)}{2\alpha {\left(\frac{1+\left(\lambda -\xi \right)\left(-2-\beta \right)}{1+\left(\lambda -\xi \right)\left(1-\beta \right)}\right)}^{m}\left[1-\right]}\left[c_{2}-\frac{\left(1-\delta \alpha \right)A_{1}+\alpha \left(1+\delta \right)A_{3}}{2A_{1}}{c_{1}}^{2}\right],$$ (20)

and

$$a_{4}\left(\beta \right)=\frac{\alpha \left(1+\delta \right)}{2\beta {\left(\frac{1+\left(\lambda -\xi \right)\left(-3-\beta \right)}{1+\left(\lambda -\xi \right)\left(1-\beta \right)}\right)}^{m}}\left[c_{3}-\frac{\left(\beta -1\right)A_{3}+2\left(1-\delta \alpha \right)A_{2}}{2A_{2}}c_{1}c_{2}+\left(\frac{3\left(\beta -1\right)\left(1-\delta \alpha \right)A_{1}A_{4}+3\alpha \left(\beta -1\right)\left(1+\delta \right)A_{3}A_{4}}{12A_{1}A_{2}}-\frac{\alpha \left(\beta -2\right)\left(1+\delta \right)A_{3}A_{4}}{12A_{1}A_{2}}-\frac{3}{12\left(1-2\delta \alpha \right)}\right){c_{1}}^{3}\right].$$ (21)

Given that h(z) has an analytic function and is confined to Ω ,

$$\left|c_{0}\right|\leq 1\;\text{and}\;\left|c_{k}\right|\leq 1-{\left|c_{0}\right|}^{2}\;\text{for}\;k>0.$$ (22)

By using Equation (22), we get

$$\left|a_{2}\left(\beta \right)\right|\leq \frac{\alpha \left(1+\delta \right)}{2\alpha {\left(\frac{1+\left(\lambda -\xi \right)\left(-1-\beta \right)}{1+\left(\lambda -\xi \right)\left(1-\beta \right)}\right)}^{m}},\left|a_{3}\left(\beta \right)\right|\leq \frac{\alpha \left(1+\delta \right)}{2\alpha {\left(\frac{1+\left(\lambda -\xi \right)\left(-2-\beta \right)}{1+\left(\lambda -\xi \right)\left(1-\beta \right)}\right)}^{m}}\left[1-\frac{\left(1-\delta \alpha \right)A_{1}+\alpha \left(1+\delta \right)A_{3}}{2A_{1}}\right]\left|a_{4}\left(\beta \right)\right|\leq \frac{\alpha \left(1+\delta \right)}{2\beta {\left(\frac{1+\left(\lambda -\xi \right)\left(-3-\beta \right)}{1+\left(\lambda -\xi \right)\left(1-\beta \right)}\right)}^{m}}\;\left[1-\frac{\left(\beta -1\right)A_{3}+2\left(1-\delta \alpha \right)A_{2}}{2A_{2}}+\frac{3\left(\beta -1\right)\left(1-\delta \alpha \right)A_{1}A_{4}+3\alpha \left(\beta -1\right)\left(1+\delta \right)A_{3}A_{4}}{12A_{1}A_{2}}-\frac{\alpha \left(\beta -2\right)\left(1+\delta \right)A_{3}A_{4}}{12A_{1}A_{2}}-\frac{3}{12\left(1-2\delta \alpha \right)}\right].$$

The proof is complete.

Using Theorem 1 and $\gamma \left(z\right)=\frac{1+z}{1-z}$ , we obtain the following.

Corollary 2. Let ${\left(f\left(z\right)\;\right)}^{\beta }\in D_{\beta }$ which is given in Equation (6). If ${\left(f\left(z\right)\;\right)}^{\beta }$ belonging to the class $L_{\xi ,\mu }^{m,\lambda }\left(1,1,\gamma \right).$ Then

$$\left|a_{2}\left(\beta \right)\right|\leq \frac{1}{\alpha {\left(\frac{1+\left(\lambda -\xi \right)\left(-1-\beta \right)}{1+\left(\lambda -\xi \right)\left(1-\beta \right)}\right)}^{m}},$$

Proof: Based on Equations (19) and (20), we have

$$\left|a_{3}\left(\beta \right)\right|\leq \frac{1}{\alpha {\left(\frac{1+\left(\lambda -\xi \right)\left(-2-\beta \right)}{1+\left(\lambda -\xi \right)\left(1-\beta \right)}\right)}^{m}}-\frac{A_{1}}{\alpha {\left(\frac{1+\left(\lambda -\xi \right)\left(-2-\beta \right)}{1+\left(\lambda -\xi \right)\left(1-\beta \right)}\right)}^{m}A_{3}}$$

and

$$\left|a_{4}\left(\beta \right)\right|\leq \frac{1}{\beta {\left(\frac{1+\left(\lambda -\xi \right)\left(-3-\beta \right)}{1+\left(\lambda -\xi \right)\left(1-\beta \right)}\right)}^{m}}.\left[1-\frac{2\left(\beta -1\right){\left(\frac{1+\left(\lambda -\xi \right)\left(-2-\beta \right)}{1+\left(\lambda -\xi \right)\left(1-\beta \right)}\right)}^{m}}{2A_{2}}+\frac{4\left(2\beta -1\right){\left(\frac{1+\left(\lambda -\xi \right)\left(-2-\beta \right)}{1+\left(\lambda -\xi \right)\left(1-\beta \right)}\right)}^{m}A_{3}}{12A_{1}A_{2}}\right]$$

We obtain the result by entering α=1 and m=0 in Corollary 2,

Corollary 3. Let f(z)∈D which is given in Equation (6). If f(z) belongs to the class $L_{\xi ,\mu }^{0,\lambda }\left(1,1,\gamma \right).$

Then

$\begin{array}{l} \left|a_{2}\left(\beta \right)\right|\leq 1\\ \left|a_{3}\left(\beta \right)\right|\leq 1, \end{array}$

and

$\left|a_{4}\left(\beta \right)\right|\leq 1.$

Theorem 4. Let $\gamma \left(z\right)=1+\alpha \left(1+\delta \right)z+\delta \alpha^{2}\left(1+\delta \right)z^{2}+\ldots ,$ where γ(z)∈D and $\overset{´}{\gamma }\left(z\right)>0.$ If ${\left(f\left(z\right)\;\right)}^{\beta }\;$ which is given in Equation (6) belongs to the class $L_{\xi ,\mu }^{m,\lambda }\left(\delta ,\alpha ,\gamma \right)$ and φ∈ℕ, then

$$\begin{array}{c} \left|a_{3}\left(\beta \right)-\varphi {a_{2}}^{2}\left(\beta \right)\right|\\ \leq \frac{\alpha \left(1+\delta \right)}{\beta {\left(\frac{1+\left(\lambda -\xi \right)\left(-2-\beta \right)}{1+\left(\lambda -\xi \right)\left(1-\beta \right)}\right)}^{m}}\max\left\{1,|(\delta \alpha +\delta )\left(\frac{\left[2\lambda +\beta -1\right]{\left(\frac{1+\left(\lambda -\xi \right)\left(-2-\beta \right)}{1+\left(\lambda -\xi \right)\left(1-\beta \right)}\right)}^{m}}{2\beta {\left(\frac{1+\left(\lambda -\xi \right)\left(-1-\beta \right)}{1+\left(\lambda -\xi \right)\left(1-\beta \right)}\right)}^{m}}\right)|\right\}.\\ \begin{array}{c} a_{3}\left(\beta \right)-\varphi {a_{2}}^{2}\left(\beta \right)=\frac{\alpha \left(1+\delta \right)c_{2}}{2\beta {\left(\frac{1+\left(\lambda -\xi \right)\left(-2-\beta \right)}{1+\left(\lambda -\xi \right)\left(1-\beta \right)}\right)}^{m}}-\frac{\left[2\beta \alpha \left(1+\delta \right)\left(1-\delta \alpha \right){\left(\frac{1+\left(\lambda -\xi \right)\left(-1-\beta \right)}{1+\left(\lambda -\xi \right)\left(1-\beta \right)}\right)}^{2m}+\left(\beta -1\right)\beta^{2}{\left(1+\delta \right)}^{2}{\left(\frac{1+\left(\lambda -\xi \right)\left(-2-\beta \right)}{1+\left(\lambda -\xi \right)\left(1-\beta \right)}\right)}^{m}\right]}{8\beta^{2}{\left(\frac{1+\left(\lambda -\xi \right)\left(-1-\beta \right)}{1+\left(\lambda -\xi \right)\left(1-\beta \right)}\right)}^{2m}{\left(\frac{1+\left(\lambda -\xi \right)\left(-2-\beta \right)}{1+\left(\lambda -\xi \right)\left(1-\beta \right)}\right)}^{m}\;}{c_{1}}^{2}\\ -\varphi \frac{\beta^{2}{\left(1+\delta \right)}^{2}{c_{1}}^{2}}{4\alpha^{2}{\left(\frac{1+\left(\lambda -\xi \right)\left(-1-\beta \right)}{1+\left(\lambda -\xi \right)\left(1-\beta \right)}\right)}^{2m}}. \end{array} \end{array}$$

Therefore

$$\left|a_{3}\left(\beta \right)-\varphi {a_{2}}^{2}\left(\beta \right)\right|=\frac{\alpha \left(1+\delta \right)c_{2}}{2\beta {\left(\frac{1+\left(\lambda -\xi \right)\left(-2-\beta \right)}{1+\left(\lambda -\xi \right)\left(1-\beta \right)}\right)}^{m}}\left[c_{2}-v{c_{1}}^{2}\right],$$

where

$$v=\frac{1}{2}\left[1-\delta \beta +\beta \left(1+\delta \right)\left(\frac{\left(2\varphi +\beta -1\right){\left(\frac{1+\left(\lambda -\xi \right)\left(-2-\beta \right)}{1+\left(\lambda -\xi \right)\left(1-\beta \right)}\right)}^{m}}{2\beta {\left(\frac{1+\left(\lambda -\xi \right)\left(-1-\beta \right)}{1+\left(\lambda -\xi \right)\left(1-\beta \right)}\right)}^{2m}}\right)\right].$$

Using Lemma 2, we obtain

$$\left|a_{3}\left(\beta \right)-\varphi {a_{2}}^{2}\left(\beta \right)\right|\leq \frac{\alpha \left(1+\delta \right)}{\beta {\left(\frac{1+\left(\lambda -\xi \right)\left(-2-\beta \right)}{1+\left(\lambda -\xi \right)\left(1-\beta \right)}\right)}^{m}}\;\text{max}\left\{1,|\delta \alpha +\alpha \left(1+\delta \right)\left(\frac{\left[2\lambda +\beta -1\right]{\left(\frac{1+\left(\lambda -\xi \right)\left(-2-\beta \right)}{1+\left(\lambda -\xi \right)\left(1-\beta \right)}\right)}^{m}}{2\beta {\left(\frac{1+\left(\lambda -\xi \right)\left(-1-\beta \right)}{1+\left(\lambda -\xi \right)\left(1-\beta \right)}\right)}^{m}}\right)|\right\}.$$

The outcome for ${\left(f\left(z\right)\;\right)}^{\beta }$ , which is provided as follows:

$$\frac{H_{\xi ,\mu }^{m,\lambda }f{\left(z\right)}^{\beta }}{{\left(\frac{1+\left(1-\beta \right)\left(\lambda -\xi \right)}{\mu +\lambda }\right)}^{m}z^{\beta }}≺\gamma \left(z^{2}\right),$$

or

$$\frac{H_{\xi ,\mu }^{m,\lambda }f{\left(z\right)}^{\beta }}{{\left(\frac{1+\left(1-\beta \right)\left(\lambda -\xi \right)}{\mu +\lambda }\right)}^{m}z^{\beta }}≺\gamma \left(z\right).$$

The proof is complete.

The results of Lemma 4 are as follows:

Theorem 5. Let $\gamma \left(z\right)=1+\alpha \left(1+\delta \right)z+\delta \alpha^{2}\left(1+\delta \right)z^{2}+\cdots ,0\leq \delta \leq 1$ and 0<β≤1. If

${\left(f\left(z\right)\;\right)}^{\beta }$ given by Equation (6) belongs to the class $L_{\xi ,\mu }^{m,\lambda }\left(\delta ,\alpha ,\gamma \right)$ , then τ∈ℂ

$$\begin{array}{l} \left|a_{3}\left(\beta \right)-\varphi {a_{2}}^{2}\left(\beta \right)\right|\\ \leq \left\{ \;\begin{array}{ll} \frac{\alpha \left(1+\delta \right)}{\beta {\left(\frac{1+\left(\lambda -\xi \right)\left(-2-\beta \right)}{1+\left(\lambda -\xi \right)\left(1-\beta \right)}\right)}^{m}}\left[\delta \alpha -\alpha \left(1+\delta \right)\left(\frac{\left[2\tau +\beta -1\right]{\left(\frac{1+\left(\lambda -\xi \right)\left(-2-\beta \right)}{1+\left(\lambda -\xi \right)\left(1-\beta \right)}\right)}^{m}}{2\beta {\left(\frac{1+\left(\lambda -\xi \right)\left(-1-\beta \right)}{1+\left(\lambda -\xi \right)\left(1-\beta \right)}\right)}^{m}}\right)\right] & \text{if}\;\tau \leq \tau_{1}\;\\ \frac{\alpha \left(1+\delta \right)}{\beta {\left(\frac{1+\left(\lambda -\xi \right)\left(-2-\beta \right)}{1+\left(\lambda -\xi \right)\left(1-\beta \right)}\right)}^{m}}, & \text{if}\;\tau_{1}\leq \tau \leq \tau_{2}\;\\ \frac{\alpha \left(1+\delta \right)}{\beta {\left(\frac{1+\left(\lambda -\xi \right)\left(-2-\beta \right)}{1+\left(\lambda -\xi \right)\left(1-\beta \right)}\right)}^{m}}\left[\alpha \left(1+\delta \right)\left(\frac{\left[2\tau +\beta -1\right]{\left(\frac{1+\left(\lambda -\xi \right)\left(-2-\beta \right)}{1+\left(\lambda -\xi \right)\left(1-\beta \right)}\right)}^{m}}{2\beta {\left(\frac{1+\left(\lambda -\xi \right)\left(-1-\beta \right)}{1+\left(\lambda -\xi \right)\left(1-\beta \right)}\right)}^{m}}\right)-\delta \alpha \right], & \text{if}\;\tau \geq \tau_{2}, \end{array}\right. \end{array}$$

Where

$$\begin{array}{c} \tau_{1}=\frac{2\alpha \left(\delta \alpha -1\right){\left(\frac{1+\left(\lambda -\xi \right)\left(-1-\beta \right)}{1+\left(\lambda -\xi \right)\left(1-\beta \right)}\right)}^{2m}-\alpha \left(1+\delta \right)\left(\beta -1\right){\left(\frac{1+\left(\lambda -\xi \right)\left(-2-\beta \right)}{1+\left(\lambda -\xi \right)\left(1-\beta \right)}\right)}^{m}}{2\alpha \left(1+\delta \right){\left(\frac{1+\left(\lambda -\xi \right)\left(-2-\beta \right)}{1+\left(\lambda -\xi \right)\left(1-\beta \right)}\right)}^{m}},\\ \tau_{2}=\frac{2\alpha \left(\delta \alpha +1\right){\left(\frac{1+\left(\lambda -\xi \right)\left(-1-\beta \right)}{1+\left(\lambda -\xi \right)\left(1-\beta \right)}\right)}^{2m}-\alpha \left(1+\delta \right)\left(\beta -1\right){\left(\frac{1+\left(\lambda -\xi \right)\left(-2-\beta \right)}{1+\left(\lambda -\xi \right)\left(1-\beta \right)}\right)}^{m}}{2\alpha \left(1+\delta \right){\left(\frac{1+\left(\lambda -\xi \right)\left(-2-\beta \right)}{1+\left(\lambda -\xi \right)\left(1-\beta \right)}\right)}^{m}}. \end{array}$$

Proof. Since ${\left(f\left(z\right)\;\right)}^{\beta }\in L_{\xi ,\mu }^{m,\lambda }\left(\delta ,\alpha ,\gamma \right)$ and γ(z) given by Equation (17), then $a_{2}\left(\beta \right)$ and $a_{3}\left(\beta \right)$ are given in Theorem 1. Furthermore,

$$\left|a_{3}\left(\beta \right)-\varphi {a_{2}}^{2}\left(\beta \right)\right|=\frac{\alpha \left(1+\delta \right)}{2\beta {\left(\frac{1+\left(\lambda -\xi \right)\left(-2-\beta \right)}{1+\left(\lambda -\xi \right)\left(1-\beta \right)}\right)}^{m}}\left[c_{2}-t{c_{1}}^{2}\right],$$

where

$$t=\frac{1}{2}\left[1-\delta +\alpha \left(1+\delta \right)\left(\frac{\left[2\tau +\beta -1\right]{\left(\frac{1+\left(\lambda -\xi \right)\left(-2-\beta \right)}{1+\left(\lambda -\xi \right)\left(1-\beta \right)}\right)}^{m}}{2\beta {\left(\frac{1+\left(\lambda -\xi \right)\left(-1-\beta \right)}{1+\left(\lambda -\xi \right)\left(1-\beta \right)}\right)}^{2m}}\right)\right].$$

Using Lemma 4, we can establish the inequality Equation (10) as follows.

$$\left|a_{3}\left(\beta \right)-\varphi {a_{2}}^{2}\left(\beta \right)\right|\leq \frac{\alpha \left(1+\delta \right)}{\beta {\left(\frac{1+\left(\lambda -\xi \right)\left(-2-\beta \right)}{1+\left(\lambda -\xi \right)\left(1-\beta \right)}\right)}^{m}}\;\left[\delta \alpha -\alpha \left(1+\delta \right)\left(\frac{\left[2\tau +\beta -1\right]{\left(\frac{1+\left(\lambda -\xi \right)\left(-2-\beta \right)}{1+\left(\lambda -\xi \right)\left(1-\beta \right)}\right)}^{m}}{2\beta {\left(\frac{1+\left(\lambda -\xi \right)\left(-1-\beta \right)}{1+\left(\lambda -\xi \right)\left(1-\beta \right)}\right)}^{m}}\right)\right].$$

When 0≤t≤1, then $\tau_{1}\leq \tau \leq \tau_{2}$ , and using Lemma 4, yields

$$\left|a_{3}\left(\beta \right)-\varphi {a_{2}}^{2}\left(\beta \right)\right|\leq \frac{\alpha \left(1+\delta \right)}{\beta {\left(\frac{1+\left(\lambda -\xi \right)\left(-2-\beta \right)}{1+\left(\lambda -\xi \right)\left(1-\beta \right)}\right)}^{m}}.$$

Assuming t≥1, then $\tau_{2}\leq \tau .$ Additionally, using Lemma 4, we get

$$\left|a_{3}\left(\beta \right)-\varphi {a_{2}}^{2}\left(\beta \right)\right|\leq \frac{\alpha \left(1+\delta \right)}{\beta {\left(\frac{1+\left(\lambda -\xi \right)\left(-2-\beta \right)}{1+\left(\lambda -\xi \right)\left(1-\beta \right)}\right)}^{m}}\left[\alpha \left(1+\delta \right)\left(\frac{\left[2\tau +\beta -1\right]{\left(\frac{1+\left(\lambda -\xi \right)\left(-2-\beta \right)}{1+\left(\lambda -\xi \right)\left(1-\beta \right)}\right)}^{m}}{2\beta {\left(\frac{1+\left(\lambda -\xi \right)\left(-1-\beta \right)}{1+\left(\lambda -\xi \right)\left(1-\beta \right)}\right)}^{m}}\right)-\delta \alpha \right]$$

Using $\gamma \left(z\right)=\frac{1+z}{1-z}$ and Theorem 5, we arrive at the following conclusion:

Corollary 6. Let $\gamma \left(z\right)=1+2z+2\;z^{2}+\cdots .\text{if}\;{\left(f\left(z\right)\;\right)}^{\beta }\;$ which is given in Equation (6) belongs to the class $L_{\xi ,\mu }^{m,\lambda }\left(\delta ,\alpha ,\gamma \right),$ then for any real complex number σ

$$\left|a_{3}\left(\beta \right)-\varphi {a_{2}}^{2}\left(\beta \right)\right|\leq \left\{ \;\begin{array}{ll} \frac{2}{\beta {\left(\frac{1+\left(\lambda -\xi \right)\left(-2-\beta \right)}{1+\left(\lambda -\xi \right)\left(1-\beta \right)}\right)}^{m}}\left[1-\left(\frac{\left[2\tau +\beta -1\right]{\left(\frac{1+\left(\lambda -\xi \right)\left(-2-\beta \right)}{1+\left(\lambda -\xi \right)\left(1-\beta \right)}\right)}^{m}}{2\beta {\left(\frac{1+\left(\lambda -\xi \right)\left(-1-\beta \right)}{1+\left(\lambda -\xi \right)\left(1-\beta \right)}\right)}^{m}}\right)\right] & \text{if}\;\tau \leq \tau_{1}\;\\ \frac{2}{\beta {\left(\frac{1+\left(\lambda -\xi \right)\left(-2-\beta \right)}{1+\left(\lambda -\xi \right)\left(1-\beta \right)}\right)}^{m}}, & \text{if}\;\tau_{1}\leq \tau \leq \tau_{2}\;\\ \frac{2}{\beta {\left(\frac{1+\left(\lambda -\xi \right)\left(-2-\beta \right)}{1+\left(\lambda -\xi \right)\left(1-\beta \right)}\right)}^{m}}\left[\left(\frac{\left[2\tau +\beta -1\right]{\left(\frac{1+\left(\lambda -\xi \right)\left(-2-\beta \right)}{1+\left(\lambda -\xi \right)\left(1-\beta \right)}\right)}^{m}}{2\beta {\left(\frac{1+\left(\lambda -\xi \right)\left(-1-\beta \right)}{1+\left(\lambda -\xi \right)\left(1-\beta \right)}\right)}^{m}}\right)-1\right], & \text{if}\;\tau \geq \tau_{2}, \end{array}\right.$$

where

$$\begin{array}{c} \tau_{1}=\frac{-\left(1-\beta \right)}{2\left(1+\delta \right)},\\ \tau_{2}=\frac{4\beta {\left(\frac{1+\left(\lambda -\xi \right)\left(-1-\beta \right)}{1+\left(\lambda -\xi \right)\left(1-\beta \right)}\right)}^{2m}-2\left(1-\beta \right){\left(\frac{1+\left(\lambda -\xi \right)\left(-2-\beta \right)}{1+\left(\lambda -\xi \right)\left(1-\beta \right)}\right)}^{m}}{4{\left(\frac{1+\left(\lambda -\xi \right)\left(-2-\beta \right)}{1+\left(\lambda -\xi \right)\left(1-\beta \right)}\right)}^{m}}. \end{array}$$

Observe that we obtain the following Corollary for and in Corollary 6.

For β=1 and m=0 , Corollary 6 yields the following.

Corollary 7. If $\gamma \left(z\right)=1+2z+2\;z^{2}+\cdots .\text{if}\;{\left(f\left(z\right)\;\right)}^{\beta }$ given by Equation (6) belongs to the class

$$L_{\xi ,\mu }^{0,\lambda }\left(1,1,\gamma \right).$$

Then for any real complex number σ

$$\left|a_{2}\left(\beta \right)-\tau {a_{1}}^{2}\left(\beta \right)\right|\leq \left\{ \begin{array}{ll} 2-4\tau & \text{if}\;\tau \leq \tau_{1},\\ 2 & \text{if}\;\tau_{1}\leq \tau \leq \\ 4\tau -2 & \text{if}\;\tau \geq \tau_{2,} \end{array}\right.\tau_{2},$$

The result of W. Ma. D. Minda [Ref. 8, Lemma 1] is obtained when $\tau_{1}=0$ and $\tau_{2}=1$ .

Corollary 8. Let $\gamma \left(z\right)=1+\alpha \left(1+\delta \right)z+\delta \alpha^{2}\left(1+\delta \right)z^{2}+\cdots ,0\leq \delta \leq 1\;$ and $0<\beta \leq 1.\text{If}{\left(f\left(z\right)\;\right)}^{\beta }$ given by Equation (6) belongs to the class $\;L_{\xi ,\mu }^{m,\lambda }\left(\delta ,\alpha ,\gamma \right),$ and $\tau_{1}\leq \tau_{3}\leq \tau_{2}.$ Let

$$\begin{array}{c} \tau_{3}=\frac{1}{2\alpha \left(1+\delta \right){\left(\frac{1+\left(\lambda -\xi \right)\left(-2-\beta \right)}{1+\left(\lambda -\xi \right)\left(1-\beta \right)}\right)}^{m}}\\ \left(2\text{βδαβ}{\left(\frac{1+\left(\lambda -\xi \right)\left(-1-\beta \right)}{1+\left(\lambda -\xi \right)\left(1-\beta \right)}\right)}^{2m}-\alpha \left(1+\delta \right)\left(1-\beta \right){\left(\frac{1+\left(\lambda -\xi \right)\left(-2-\beta \right)}{1+\left(\lambda -\xi \right)\left(1-\beta \right)}\right)}^{m}\right). \end{array}$$

If $\;\tau_{1}\leq \tau \leq \tau_{3},$ then

$$\leq \frac{\beta \left(1+\delta \right)}{2\beta {\left(\frac{1+\left(\lambda -\xi \right)\left(-2-\beta \right)}{1+\left(\lambda -\xi \right)\left(1-\beta \right)}\right)}^{m}}.$$ (23)

If $\tau_{3}\leq \tau \leq \tau_{2}$ , then

$$\begin{array}{c} \left|a_{3}\left(\beta \right)-\tau {a_{2}}^{2}\left(\beta \right)\right|+\frac{2\beta {\left(\frac{1+\left(\lambda -\xi \right)\left(-1-\beta \right)}{1+\left(\lambda -\xi \right)\left(1-\beta \right)}\right)}^{2m}}{\alpha \left(1+\delta \right){\left(\frac{1+\left(\lambda -\xi \right)\left(-2-\beta \right)}{1+\left(\lambda -\xi \right)\left(1-\beta \right)}\right)}^{m}}\\ \times \left[1-\delta \alpha +\alpha \left(1+\delta \right)\left(\frac{\left[2\tau +\beta -1\right]{\left(\frac{1+\left(\lambda -\xi \right)\left(-2-\beta \right)}{1+\left(\lambda -\xi \right)\left(1-\beta \right)}\right)}^{m}}{2\beta {\left(\frac{1+\left(\lambda -\xi \right)\left(-1-\beta \right)}{1+\left(\lambda -\xi \right)\left(1-\beta \right)}\right)}^{m}}\right)\right]{\left|a_{2}\left(\beta \right)\right|}^{2}\\ \begin{array}{c} \left|a_{3}\left(\beta \right)-\tau {a_{2}}^{2}\left(\beta \right)\right|+\frac{2\beta {\left(\frac{1+\left(\lambda -\xi \right)\left(-1-\beta \right)}{1+\left(\lambda -\xi \right)\left(1-\beta \right)}\right)}^{2m}}{\alpha \left(1+\delta \right){\left(\frac{1+\left(\lambda -\xi \right)\left(-2-\beta \right)}{1+\left(\lambda -\xi \right)\left(1-\beta \right)}\right)}^{m}}\\ \times \left[1-\delta \alpha +\alpha \left(1+\delta \right)\left(\frac{\left[2\tau +\beta -1\right]{\left(\frac{1+\left(\lambda -\xi \right)\left(-2-\beta \right)}{1+\left(\lambda -\xi \right)\left(1-\beta \right)}\right)}^{m}}{2\beta {\left(\frac{1+\left(\lambda -\xi \right)\left(-1-\beta \right)}{1+\left(\lambda -\xi \right)\left(1-\beta \right)}\right)}^{m}}\right)\right]{\left|a_{2}\left(\beta \right)\right|}^{2}\\ \leq \frac{\beta \left(1+\delta \right)}{2\beta {\left(\frac{1+\left(\lambda -\xi \right)\left(-2-\beta \right)}{1+\left(\lambda -\xi \right)\left(1-\beta \right)}\right)}^{m}} \end{array} \end{array}$$ (24)

Proof. For

$$\begin{array}{c} \left|a_{3}\left(\beta \right)-\tau {a_{2}}^{2}\left(\beta \right)\right|+\left(\tau -\tau_{1}\right){\left|a_{2}\left(\beta \right)\right|}^{2}=\\ \left(\tau -\frac{1}{2\alpha \left(1+\delta \right){\left(\frac{1+\left(\lambda -\xi \right)\left(-2-\beta \right)}{1+\left(\lambda -\xi \right)\left(1-\beta \right)}\right)}^{m}}\left[2\beta \left(\delta \alpha -1\right){\left(\frac{1+\left(\lambda -\xi \right)\left(-1-\beta \right)}{1+\left(\lambda -\xi \right)\left(1-\beta \right)}\right)}^{2m}+\alpha \left(1+\delta \right)\left(\beta -1\right){\left(\frac{1+\left(\lambda -\xi \right)\left(-2-\beta \right)}{1+\left(\lambda -\xi \right)\left(1-\beta \right)}\right)}^{m}\right]\right)\\ \begin{array}{c} \times \frac{\beta^{2}{\left(1+\delta \right)}^{2}}{{\left(\frac{1+\left(\lambda -\xi \right)\left(-1-\beta \right)}{1+\left(\lambda -\xi \right)\left(1-\beta \right)}\right)}^{2m}}{\left|c_{1}\right|}^{2}+\frac{\alpha \left(1+\delta \right)}{2\alpha {\left(\frac{1+\left(\lambda -\xi \right)\left(-2-\beta \right)}{1+\left(\lambda -\xi \right)\left(1-\beta \right)}\right)}^{m}}\left[c_{2}-t{c_{1}}^{2}\right]\\ =\frac{\alpha \left(1+\delta \right)}{2\alpha {\left(\frac{1+\left(\lambda -\xi \right)\left(-2-\beta \right)}{1+\left(\lambda -\xi \right)\left(1-\beta \right)}\right)}^{m}}\left[c_{2}-t{c_{1}}^{2}\left(\beta \right)+t{\left|c_{1}\left(\beta \right)\right|}^{2}\right]. \end{array} \end{array}$$

It follows that using Lemma 4 yields

$$\left|a_{3}\left(\beta \right)-\tau {a_{2}}^{2}\left(\beta \right)\right|+\left(\tau -\tau_{1}\right){\left|a_{2}\left(\beta \right)\right|}^{2}\leq \frac{\alpha \left(1+\delta \right)}{2\alpha {\left(\frac{1+\left(\lambda -\xi \right)\left(-2-\beta \right)}{1+\left(\lambda -\xi \right)\left(1-\beta \right)}\right)}^{m}},$$

This is the inequality of Equation (24). For

$$\begin{array}{c} \left|a_{3}\;\left(\beta \right)-\tau {a_{2}}^{2}\left(\beta \right)\right|+\left(\tau -\tau_{1}\right){\left|a_{2}\left(\beta \right)\right|}^{2}\\ =\left(\frac{2\beta \left(\alpha \delta +1\right){\left(\frac{1+\left(\lambda -\xi \right)\left(-1-\beta \right)}{1+\left(\lambda -\xi \right)\left(1-\beta \right)}\right)}^{2m}}{2\beta \left(1+\delta \right){\left(\frac{1+\left(\lambda -\xi \right)\left(-2-\beta \right)}{1+\left(\lambda -\xi \right)\left(1-\beta \right)}\right)}^{m}}-\frac{\alpha \left(1+\delta \right)\left(\beta -1\right){\left(\frac{1+\left(\lambda -\xi \right)\left(-2-\beta \right)}{1+\left(\lambda -\xi \right)\left(1-\beta \right)}\right)}^{m}}{\beta \left(1+\delta \right){\left(\frac{1+\left(\lambda -\xi \right)\left(-2-\beta \right)}{1+\left(\lambda -\xi \right)\left(1-\beta \right)}\right)}^{m}}-\tau \right)\\ \begin{array}{c} \times \frac{\alpha^{2}{\left(1+\delta \right)}^{2}}{4\beta {\left(\frac{1+\left(\lambda -\xi \right)\left(-1-\beta \right)}{1+\left(\lambda -\xi \right)\left(1-\beta \right)}\right)}^{2m}}{\left|c_{1}\left(\beta \right)\right|}^{2}+\frac{\alpha \left(1+\delta \right)}{2\beta {\left(\frac{1+\left(\lambda -\xi \right)\left(-2-\beta \right)}{1+\left(\lambda -\xi \right)\left(1-\beta \right)}\right)}^{m}}\left[c_{2}-t{c_{1}}^{2}\right]\\ =\frac{\alpha \left(1+\delta \right)}{2\beta {\;(\frac{1+\left(\lambda -\xi \right)\left(-2-\beta \right)}{1+\left(\lambda -\xi \right)\left(1-\beta \right)})}^{m}}\left[\left|c_{2}-t{c_{1}}^{2}\right|+t{\left|c_{1}\right|}^{2}\right]. \end{array} \end{array}$$

Using Lemma 4, the following results

$$\left|a_{3}\left(\beta \right)-\tau {a_{2}}^{2}\left(\beta \right)\right|+\left(\tau -\tau_{1}\right){\left|a_{2}\left(\beta \right)\right|}^{2}\leq \frac{\alpha \left(1+\delta \right)}{2\alpha {\left(\frac{1+\left(\lambda -\xi \right)\left(-2-\beta \right)}{1+\left(\lambda -\xi \right)\left(1-\beta \right)}\right)}^{m}},$$

which is the inequality of Equation (24).

## Conclusions

In this study, we have investigated a subclass of Bazilevič-type functions associated with the class of univalent functions and examined its analytic properties using differential subordination and differential operators. By applying these techniques, new coefficient estimates and Fekete–Szegö inequalities were derived, providing sharper and more general results than several existing findings in the literature. The obtained results demonstrate that the parameters defining the subclass play a significant role in determining the coefficient behavior and geometric characteristics of the associated analytic functions. The study contributes to the deeper understanding of the coefficient structure of Bazilevič-type functions and extends the theoretical framework of univalent function theory. Furthermore, the approach used in this work may be applied to other subclasses of analytic or multivalent functions to establish similar inequalities and functional relationships. Future research may focus on exploring additional subclasses generated by modified differential operators or by incorporating complex parameters that enhance the geometric interpretation of these functions in applied fields such as fluid dynamics and conformal mapping.

## Ethical considerations

This study did not involve human participants or animals, and therefore, ethical approval was not required.

## Data Availability

No data are associated with this article. This manuscript is paper in the field of Complex Analysis. Therefore, no specific reporting guidelines (such as CONSORT, PRISMA, or STROBE, which apply to empirical or experimental studies) are applicable. The paper follows standard mathematical reporting practices: all definitions, theorems, and proofs are presented with full logical rigor, and all symbols and notations are clearly defined upon first use. The structure and exposition conform to conventional mathematical standards for reproducibility and clarity.
