# Supplementary material for: A Bazilevic-type function associated with the class of univalent functions with study Coefficient Bounds and Fekete-Szego inequalities
Source: F1000Res. 2026 Sep 15;15:10. Originally published 2026 Jan 6. [Version 2] doi: 10.12688/f1000research.172490.2 (PMC13675624; doi:10.12688/f1000research.172490.2)
Supplement: Peer review history

# Reviewer report: Reviewer response for version 2

**DOI:** 10.5256/f1000research.201230.r523114

**Published:** 2026-10-16

**Authors:** Majeed Ahmad Yousif

**Affiliations:** 1 Department of Mathematics, College of Education, University of Zakho, Duhok, Iraq

The manuscript investigates coefficient bounds and Fekete–Szegö inequalities for a subclass of Bazilevič-type univalent functions defined through a differential operator and subordination approach. The topic is relevant to geometric function theory, and the paper develops explicit estimates for several coefficients together with corresponding Fekete–Szegö-type results. The manuscript also provides special cases and corollaries that connect the proposed class with previously studied subclasses.

 The mathematical contribution appears potentially useful; however, the presentation should be improved before final acceptance. In particular, the authors should carefully check mathematical notation, grammatical structure, consistency of parameters, and the clarity of several equations and proofs. Some statements in the Methods section require more precise mathematical wording, and the motivation for introducing the particular differential operator and new subclass should be strengthened. The authors should also explain more explicitly how the main theorems extend or improve existing coefficient estimates rather than only presenting special cases. The obtained results demonstrate parameter-dependent coefficient bounds and extensions of earlier Bazilevič-type results.

Is the work clearly and accurately presented and does it cite the current literature?

Yes

If applicable, is the statistical analysis and its interpretation appropriate?

No

Are all the source data underlying the results available to ensure full reproducibility?

Yes

Is the study design appropriate and is the work technically sound?

Yes

Are the conclusions drawn adequately supported by the results?

Yes

Are sufficient details of methods and analysis provided to allow replication by others?

Yes

Reviewer Expertise:

Numerical anlysis

I confirm that I have read this submission and believe that I have an appropriate level of expertise to confirm that it is of an acceptable scientific standard, however I have significant reservations, as outlined above.

## References

[1] : Analytic function classes defined by Mittag–Leffler inspired Poisson-type series. *Alexandria Engineering Journal* .2026;136: 10.1016/j.aej.2026.01.00162-72 10.1016/j.aej.2026.01.001

[2] : New horizons in analytic function classes induced by the Erdélyi–Kober fractional integral operators. *An International Journal of Optimization and Control: Theories & Applications (IJOCTA)* .2025;16(1) : 10.36922/IJOCTA025290127 10.36922/IJOCTA025290127

[3] : Representation of Bi-Univalent Functions to Lucas Balancing Polynomials with Geometric Properties and Coefficient Bounds. *European Journal of Pure and Applied Mathematics* .2025;18(3) : 10.29020/nybg.ejpam.v18i3.6294 10.29020/nybg.ejpam.v18i3.6294

# Reviewer report: Reviewer response for version 2

**DOI:** 10.5256/f1000research.201230.r521486

**Published:** 2026-09-30

**Authors:** Gangadharan Murugusundaramoorthy

**Affiliations:** 1 Vellore Institute of Technology, Vellore, India

1) Define clearly the operator

 State the limits of parameters introduced here, like \lambda, \mu, \zeta, and m .

 2) H_1: What do you mean by this? What about other parameters? Why are you defining the linear operator like this? Clear explanation to be added Or cite the base paper for defining the linear operator.

 3) In equation (10), \gamma(z) is given without the range of parameters \alpha and \delta. Also \gamma^{prime}(z) > 0.

Is the work clearly and accurately presented and does it cite the current literature?

Yes

If applicable, is the statistical analysis and its interpretation appropriate?

Not applicable

Are all the source data underlying the results available to ensure full reproducibility?

Yes

Is the study design appropriate and is the work technically sound?

Yes

Are the conclusions drawn adequately supported by the results?

Yes

Are sufficient details of methods and analysis provided to allow replication by others?

Yes

Reviewer Expertise:

COMPLEX ANALYSIS GEOMETRIC FUNCTION THEORY-30C45

I confirm that I have read this submission and believe that I have an appropriate level of expertise to confirm that it is of an acceptable scientific standard, however I have significant reservations, as outlined above.

# Reviewer report: Reviewer response for version 1

**DOI:** 10.5256/f1000research.190223.r459388

**Published:** 2026-03-10

**Authors:** Georgia Irina Oros

**Affiliations:** 1 University of Oradea, Oradea, Romania

As the Abstract adequately describes, the paper investigates a specific subclass of Bazilevič-type functions, utilizing differential operators and subordination techniques to establish Fekete–Szegö inequalities and coefficient bounds. The methods used for developing the new results are classical in Geometric Function Theory, particularly differential subordination, but well used for giving complete and correct proofs for the new results. The motivation of the study is well argued in Introduction. The history of the study is given supported by well chosen and properly cited references. However, it is suggested to add a few references of recent studies from 2025, 2025 on Bazilevič-type functions. The paper is adequately structured and all the necessary information is offered in the Methods part. It is advised to highlight the known results mentioned by adding the reference in brackets, next to the definitions or lemmas. There is no reference given for Lemma 2, 3 and 4. Please indicate where they were taken from. The new outcome is established in the Results and Discussion part. The proofs are complete and correct. This is reinforced by the mention of previously obtained results that are generalized by the original outcome of this study that can be obtained by taking specific values of the parameters involved in the newly proved theorems. The corollaries that follow the new results are given for functions with particular geometric properties which makes those corollaries interesting and inspiring for applications.  

 Overall, the paper is well written and presents interesting new results.

Is the work clearly and accurately presented and does it cite the current literature?

Yes

If applicable, is the statistical analysis and its interpretation appropriate?

Not applicable

Are all the source data underlying the results available to ensure full reproducibility?

Yes

Is the study design appropriate and is the work technically sound?

Yes

Are the conclusions drawn adequately supported by the results?

Yes

Are sufficient details of methods and analysis provided to allow replication by others?

Yes

Reviewer Expertise:

Geometric Function Theory

I confirm that I have read this submission and believe that I have an appropriate level of expertise to confirm that it is of an acceptable scientific standard.

# Response

**Published:** 2026-03-25

**Authors:** Mays *Saleh

We sincerely thank the reviewer for their positive and encouraging evaluation of our manuscript.

 We are grateful for your confirmation that the work is clearly presented, technically sound, and supported by appropriate methodology and analysis. We also appreciate your acknowledgment of the adequacy of the data, reproducibility, and the validity of the conclusions.

 Your expertise in Geometric Function Theory and your supportive assessment are highly valued.

 Thank you again for your time and thoughtful review.

# Reviewer report: Reviewer response for version 1

**DOI:** 10.5256/f1000research.190223.r459389

**Published:** 2026-03-09

**Authors:** Gangadharan Murugusundaramoorthy

**Affiliations:** 1 Vellore Institute of Technology, Vellore, India

- In page 4, after line 5, by applying Equation (5) binomial expansion, we get -------- to be checked and ^\beta to be removed May english corrections
- Notational mistakes. Certain things are not defined like H and H_1
- The linear transformation in (7) is not defined properly.
- References are given properly in standard style format
- Results are correct
- Methodology techniques are okay.
- The definition of the operator is to be given clearly.
- After incorporating these points I will recommend this article for indexing.

Is the work clearly and accurately presented and does it cite the current literature?

Yes

If applicable, is the statistical analysis and its interpretation appropriate?

Not applicable

Are all the source data underlying the results available to ensure full reproducibility?

Yes

Is the study design appropriate and is the work technically sound?

Yes

Are the conclusions drawn adequately supported by the results?

Yes

Are sufficient details of methods and analysis provided to allow replication by others?

Yes

Reviewer Expertise:

COMPLEX ANALYSIS GEOMETRIC FUNCTION THEORY-30C45

I confirm that I have read this submission and believe that I have an appropriate level of expertise to confirm that it is of an acceptable scientific standard, however I have significant reservations, as outlined above.

# Response

**Published:** 2026-03-25

**Authors:** Mays *Saleh

We sincerely thank the reviewer for their careful reading, valuable comments, and constructive suggestions. Their feedback has helped us significantly improve the clarity, accuracy, and presentation of the manuscript. All comments have been carefully considered and addressed in the revised version.

  

- In page 4, after line 5, by applying Equation (5) binomial expansion, we get — to be checked and to be removed.

Response:

 Thank you for your careful reading and helpful comment. The expression on page 4 (after line 5) has been carefully checked and corrected.

- By applying the binomial expansion to Equation (5), the expression is revised as follows, and the unnecessary term .

Notational mistakes. Certain things are not defined like H and H_1.

 Response:

 Thank you for pointing this out. All notational inconsistencies have been carefully revised.

- The linear transformation in (7) is not defined properly.

Response:

 We appreciate this observation. Equation (7) has been revised, and the linear transformation is now properly defined with full clarification.

- The definition of the operator is to be given clearly.

Response:

 Thank you for this important remark. The operator definition has been rewritten and clarified to improve readability and mathematical rigor.

- Results are correct.

Response:

 We appreciate the reviewer’s confirmation of the correctness of the results.

- Methodology techniques are okay.

Response:

 We thank the reviewer for acknowledging the appropriateness of the methodology.

  

- After incorporating these points, I will recommend this article for indexing.

Response:

 We are very grateful for this encouraging statement. All suggested corrections have been fully incorporated into the revised manuscript.

- Final Remark

We sincerely thank the reviewers again for their valuable time and insightful comments, which have greatly improved the quality of our work.
